# An epidemiological study on early orthodontic treatment need among eastern Saudi Arabian children in the mixed dentition stage

**DOI:** 10.1038/s41598-024-54381-6

**Published:** 2024-02-19

**Authors:** Guna Shekhar Madiraju, Yousef Majed Almugla, Rohini Mohan, Basil Mohammed Alnasser

**Affiliations:** 1 Department of Preventive Dental Sciences, Faculty in Pediatric Dentistry, College of Dentistry, King Faisal University, 31982 Al Ahsa, Saudi Arabia; 2 Department of Preventive Dental Sciences, Faculty in Orthodontics, College of Dentistry, King Faisal University, 31982 Al Ahsa, Saudi Arabia; 3 Community Dental Services, Port Talbot Research Centre, Swansea Bay University Health Board, Port Talbot, UK; 4 Dental Intern, College of Dentistry, King Faisal University, 31982 Al Ahsa, Saudi Arabia

**Keywords:** Health care, Dentistry, Public health

## Abstract

Estimation of early orthodontic treatment need among children is essential for planning orthodontic interventions in the mixed dentition stages thereby reducing the burden in a publicly funded healthcare system. The present study aimed to assess the early orthodontic treatment need among children with mixed dentition in the Eastern Saudi Arabia. A descriptive cross-sectional study was conducted among Saudi children visiting the outpatient clinics in a University dental setting, and data were collected based on Index for preventive and interceptive orthodontic need (IPION). Descriptive statistics, chi-square test and Fisher’s exact test were used for data analysis with statistical significance set at p < 0.05. The category of ‘no treatment need’ accounted for 11.3% while ‘moderate treatment need’ and ‘definite treatment need’ categories accounted for 29.3% and 59.4% respectively. There was no statistical difference between males and females in the distribution of the three categories of treatment need (p = 0.513). This study demonstrated a very high need for early orthodontic treatment among Saudi children in the mixed dentition stage. Emphasis should be placed on increased awareness and benefits of seeking early orthodontic treatment involving preventive and interceptive procedures in the mixed dentition.

## Introduction

Early orthodontic treatment in the mixed dentition aids in reducing or eliminating the severity of developing malocclusion, which further could reduce the financial burden associated with orthodontic treatment at later stages^1–3^. Previous studies conducted in different regions of Saudi Arabia had demonstrated orthodontic treatment need ranging from 4.4 to 30.9%^4–6^. A recent study conducted by Alyami et al. reported 47.6% prevalence of malocclusion among Saudi adolescents from the southern region and the need for orthodontic treatment was 19.46%^7^. The prevalence and severity of malocclusion have risen during the last decades and there has been an increase in the demand for orthodontic treatment in the Saudi population^8^. Early orthodontic treatment has several benefits including better use of growth potential, more treatment options, reduced need for extractions, better patient compliance and satisfaction. Assessment of early orthodontic treatment need aids in planning preventive and interceptive measures, which is crucial for proper utilization of resources in publicly funded healthcare system.

Several indices have been used in different populations to evaluate orthodontic treatment needs such as dental aesthetic index^9^, Swedish National Board of Health and Welfare index^10^, and Index for Orthodontic Treatment Need^4–6,11^. Index for preventive and interceptive orthodontic needs (IPION) has been developed to determine the treatment needs specifically in mixed dentition^1^. IPION was developed as two indices, the IPION-6 and IPION-9, based on the chronologic age of the child, wherein IPION-9 index identifies children who would most likely benefit from early orthodontic treatment including preventive and/or interceptive procedures. The overall score calculated using IPION reflects the child’s compliance for early orthodontic treatment.

Collection of epidemiological information on a regular basis is vital to assess the needs of a population. Eastern province of Saudi Arabia is the largest in terms of area and the third most populous region. Moreover, orthodontic treatment in Saudi Arabia is mostly implemented through resources from public health system funding. Data available on the early orthodontic treatment need among children from Eastern Saudi Arabia is scarce in the literature. Such data might be helpful in adopting policy changes related to implementing orthodontic interventions at early stages of dentition. The purpose of the study was to evaluate early orthodontic treatment need in a sample of children from the Eastern province of Saudi Arabia.

## Methods

The present cross-sectional study was conducted as part of a series of investigations analyzing the early orthodontic treatment need among Saudi children in Al Ahsa, Eastern province of Saudi Arabia. The study protocol was reviewed and approved by the Institutional ethical and review board of the Deanship of Scientific Research, King Faisal University, Al Ahsa, Saudi Arabia (Ref: KFU-REC/2020-11-24) and the study was conducted in accordance with the ethical standards laid down in 1964 Declaration of Helsinki. Written informed consent was obtained from parents and/or guardians of all the study participants. The study is reported in line with strengthening the Reporting of Observational Studies in Epidemiology (STROBE) recommendations for cross-sectional studies.

### Study design

This descriptive study involved Saudi children aged 8–9 years with mixed dentition, and the IPION-9 criteria was used on those children whose permanent maxillary central incisors were visible upon examination, even if their chronological age indicated that they were not nine-years old. Exclusion criteria included subjects who were non-cooperative, had cleft lip cleft palate or other craniofacial syndromes; no previous or current orthodontic treatment; and those having incomplete information. The sample size estimated at 50% prevalence with α of 5% and a power of 95% required a minimum sample of 385 subjects for this study. Convenience sampling technique was used to enroll the study sample. During the study period from November 2019 to June 2022, random dates were chosen, and screening of children was conducted. Children visiting the dental clinics in a University setting for initial examination, restorative and/or oral hygiene needs were selected based on their dental records on the day of their visit. Child age at the last birthday was considered as the age at the time of examination. Participation in the study was voluntary and no child was refused a consultation.

### Data collection

Each subject was examined in a dental chair in an upright position using disposable surgical gown and gloves, face mask, face shield, sterile mouth mirror, and wooden tongue depressor. Community periodontal index probes (CPITN) were used to measure the parameters in millimeters, where needed. Infection control procedures as outlined by the Centers for Disease Control and Prevention were used^12^. IPION was developed as two indices including IPION-6 and the IPION-9. The present study utilized IPION-9, which constitutes 16 components, and each component has its own weighing score, which are multiplied by their weighting factors and added to get an overall score for each subject. Based on the overall score, the subjects were organized into three categories of treatment needs, according to the original study by Coetzee^1^. The scores of 0–5 were considered ‘no treatment need’, the scores of 6–14 were considered ‘moderate treatment need’ and the scores of 15 or higher were considered ‘definite treatment need.

Two experienced and calibrated examiners evaluated all the cases together, and the disagreements were discussed until consensus was reached. Prior to the study, the examiners were calibrated by examining and recording the scores of 30 children, who were later excluded from the final sample. These children were re-examined after a two-week interval to establish inter- and intra-examiner reliability. The intra-examiner and inter-examiner agreements evaluated using the weighted kappa statistic were found to be 0.91 and 0.89, respectively.

### Data analysis

The data were subjected to statistical analysis by using the Statistical Package for the Social Sciences (SPSS version 20, IBM Corp., USA). Descriptive statistics and frequencies were calculated for the presentation of the results. The Pearsons chi-square test or fisher’s exact tests were used to detect any statistical differences between the gender and the components of IPION, and treatment need categories. Proportion test was used to compare ‘definite treatment need’ among males and females. Statistical significance was considered with p < 0.05.

## Results

A total number of 478 children participated in the study (206 males and 272 females) and their age ranged from 7 years 10 months to 9 years 3 months. Eleven out of 489 cases in whom any of the IPION-9 components could not be measured were excluded from the final sample. The distribution of components of IPION-9 among the study sample by gender are presented in Tables 1 and 2. Proximal caries of the first primary molar (D) had the highest prevalence in this study (77%) followed by second primary molar (70.3%). Around 75.4% of the sample had caries affected first permanent molars. Loss of second primary molars were seen in 51.9% of the sample. The most prevalent component was abnormal first permanent molar relation (54%), followed by overbite > 2/3rd (50.2%), and overjet > 3.1 mm (44.3%) respectively. Caries was determined as cavitated lesions, and permanent incisors were recorded as absent regardless of the cause. Ectopic eruption of first permanent molars and submerged primary first molar were not seen in this study.

**Table 1:** Distribution of primary & soft tissue components of IPION-9 by gender.

IPION-9 components	Male (n = 206)	Female (n = 272)	Total	*p*-value**
‘C’ proximal caries				
No	110 (53.4)	150 (55.1)	260 (54.4)	0.292
1 Tooth	54 (26.2)	56 (20.6)	110 (23.0)	
> 1 Tooth	42 (20.4)	66 (24.3)	108 (22.6)	
‘D’ proximal caries				
No	40 (19.4)	70 (25.7)	110 (23)	0.005
1 Tooth	52 (25.2)	92 (33.8)	144 (30.1)	
> 1 Tooth	114 (55.3)	110 (40.4)	224 (46.9)	
‘E’ proximal caries				
No	54 (26.2)	88 (32.4)	142 (29.7)	0.240
1 Tooth	64 (31.1)	86 (31.6)	150 (31.4)	
> 1 Tooth	88 (42.7)	98 (36.0)	186 (38.9)	
FPM caries				
No	50 (24.3)	68 (25.0)	118 (24.7)	0.799
1 Tooth	56 (27.2)	80 (29.4)	136 (28.5)	
> 1 Tooth	100 (48.5)	124 (45.6)	224 (46.9)	
Early loss of ‘E’				
No	100 (48.5)	130 (47.8)	230 (48.1)	0.034
1 Tooth	74 (35.9)	76 (27.9)	150 (31.4)	
> 1 Tooth	32 (15.5)	66 (24.3)	98 (20.5)	
Submerged ‘E’				
No	202 (98.1)	270 (99.3)	472 (98.7)	0.410^†^
Yes	04 (1.9)	02 (0.7)	06 (1.3)	
Incompetent lips	192 (93.2)	240 (88.2)	432 (90.4)	0.084^†^
No	14 (6.8)	32 (11.8)	46 (9.6)	
Yes (< 4 mm space between lips at rest)				

*C* primary canine, *D* primary first molar, *E* primary second molar, *FPM* first permanent molar.

*p-value: Chi-square test.

^†^Fishers exact test; numbers in parentheses indicate percentage (%).

**Table 2:** Distribution of anterior, posterior & occlusal components of IPION-9 by gender.

IPION-9 components	Male (n = 206)	Female (n = 272)	Total	*p*-value**
Overjet (mm)				
0–3.0	130 (63.1)	138 (50.0)	266 (55.6)	0.016
3.1–5.0	52 (25.2)	90 (33.1)	142 (29.7)	
5.1–7.0	24 (11.7)	46 (16.9)	70 (14.6)	
Overbite				
< 1/3rd coverage	28 (13.6)	38 (13.9)	66 (13.80)	0.692
1/3–2/3rd coverage	82 (39.8)	98 (36.03)	180 (37.7)	
> 2/3 coverage	96 (46.6)	136 (50.0)	232 (48.5)	
Open bite (mm)				
No	197 (95.6)	259 (95.2)	456 (95.39)	0.970
< 1.0	03 (1.46)	04 (1.47)	07 (1.46)	
1.0–2.0	06 (2.91)	09 (3.30)	15 (3.14)	
Anterior crossbite				
No	184 (89.3)	232 (85.3)	416 (87)	0.217^†^
Yes (crossbite tendency)	22 (10.7)	40 (14.7)	62 (13)	
Posterior crossbite				
No	194 (94.2)	256 (94.1)	450 (94.1)	1.000^†^
Yes	12 (5.8)	16 (5.9)	28 (5.9)	
FPM relation*				
Normal	92 (44.7)	128 (47.1)	220 (46)	0.643^†^
Abnormal	114 (55.3)	144 (52.9)	258 (54)	
Upper molar rotation				
No	196 (95.1)	248 (91.2)	444 (92.9)	0.107^†^
Yes	10 (4.9)	24 (8.8)	34 (7.1)	
Lower molar tipping				
No	162 (78.6)	208 (76.5)	370 (77.4)	0.583^†^
Yes	44 (21.4)	64 (23.5)	108 (22.6)	
Diastema > 2mm				
No	194 (94.2)	248 (91.2)	442 (92.5)	0.293^†^
Yes	12 (5.8)	24 (8.8)	36 (7.5)	
Supernumerary teeth				
No	202 (98.1)	270 (99.3)	472 (98.7)	0.409^†^
Yes	04 (1.9)	02 (0.7)	06 (1.3)	
Absent permanent incisors				
No	204 (99)	266 (97.8)	470 (98.3)	0.475^†^
Yes	02 (1)	06 (2.2)	08 (1.7)	
Active frenum				
No	202 (98.1)	250 (91.9)	452 (94.6)	0.003^†^
Yes	04 (1.9)	22 (8.1)	26 (5.4)	

*FPM* first permanent molar.

*Normal first permanent molar relationship is defined as the Class I molar relationship ± 2 mm on both sides.

**p-value: Chi-square test.

^†^Fishers exact test; numbers in parentheses indicate percentage (%).

Posterior crossbite assessment included either with (score 10) or without (score 1) functional lateral shift of the mandible during closure. Among the 28 (5.9%) children with posterior crossbite, 26 (11 males; 15 females) were assigned to score-1, while the remaining 2 children (one each in male and female) were assigned to score-10. Females showed greater percentage of occlusal traits compared to their counterparts. No significant differences were noted between the gender and the components of IPION-9, except for the proximal caries in first primary molars (p = 0.005), early loss of second primary molar (p = 0.034), overjet of more than 3 mm (p = 0.016), and active frenum (p = 0.004).

**Figure 1:**
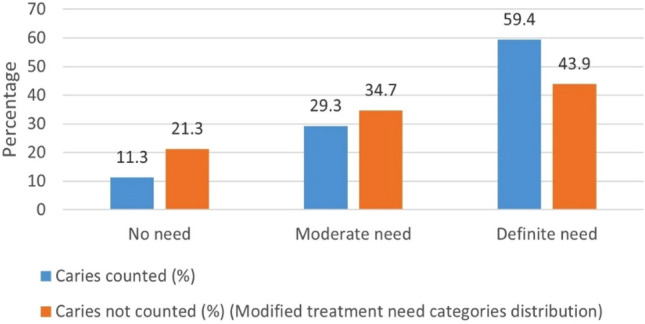
Distribution of children, according to the IPION-9 treatment need categories.

Based on the overall scores in the study sample, the distribution of treatment need categories was determined by calculating frequencies and percentages. Figure 1 illustrates the percentage distribution of IPION-9 treatment need in the study population. The category of ‘no treatment need’ accounted 11.3%, while ‘moderate need’ and ‘definite need’ categories accounted for 29.3% and 59.4% respectively. When the weighted points accumulated for caries are deducted from the overall scores for each subject, the impact of caries was identified by a modified treatment need category distribution. Of the total study sample, modified treatment need distribution showed that 21.3% formed the category of ‘no treatment need’, followed by 34.7% in ‘moderate need’ and 43.9% in ‘definite need’ of orthodontic treatment.

Gender distribution of the treatment need according to the IPION-9 showed that 57.3% of males and 61% of females were in the category of ‘definite need’; 32% of males were in ‘moderate need’ and 10.7% were in ‘no need’ category. Meanwhile, ‘moderate need’ and ‘no need’ treatment for females were 27.2% and 11.8%, respectively. Chi-square test showed that gender distribution of the IPION-9 treatment need was not statistically significant (χ^2^ = 1.334; p = 0.513) (Table 3). A proportion test was used to compare ‘definite treatment need’ among males and females. Total proportion of ‘definite treatment need’ category was 59.4%, of which females presented a slightly higher percentage of treatment need (61.0%) than in males (57.3%), whilst it was not statistically significant (p = 0.408).

**Table 3:** Distribution of orthodontic treatment need based on gender.

Treatment need	Male n (%)	Female n (%)	Total n (%)	*p-*value* Chi-square test
Definite need	118 (57.3)	166 (61.0)	284 (59.4)	0.513
Moderate need	66 (32.0)	74 (27.2)	140 (29.3)	
No need	22 (10.7)	32 (11.8)	54 (11.3)	
Total	206 (100)	272 (100)	478 (100)	

## Discussion

The present study investigated the early orthodontic treatment need in a sample of Saudi children from the Eastern region of Saudi Arabia and found that 88.7% of the sample presented either moderate or definite preventive and interceptive orthodontic treatment need, which implies high treatment need percentage in the present sample. Previous studies^4–6,11,13^ conducted in Saudi Arabia to evaluate orthodontic treatment need have shown variable results for definite treatment need ranging from 4.4 to 52.5%, which is lower than that found in the present study (59.4%). Yilmaz et al.^14^ had reported that 45.9% of 7–8-year-old and 56.9% of 9–10-year-old Turkish children showed definite orthodontic treatment need. Salim et al.^15^ in a study on Syrian refugee children in Jordan used dental health component of Index for Orthodontic Treatment Need (IOTN-DHC) and found 67.7% prevalence of moderate to severe need for orthodontic treatment. Bourzgui et al.^10^ had reported a higher need (86.3%) for orthodontic treatment in 8–10-year-old Moroccan schoolchildren. Another study conducted in Saudi Arabia using IOTN-DHC reported that 30.9% of 8- to 9-year-old Saudi children possessed definite need for orthodontic treatment^5^. However, comparison of these findings should be made with caution, as they were conducted in a different and/or wider range of age groups using IOTN index.

Studies reporting on the orthodontic treatment need using IPION index are scarce in the literature. Karaiskos et al.^16^ in a study on Canadian children reported that 37.1% of children in IPION-9 group scored ‘5’ or higher, indicating the need for preventive and interceptive orthodontic treatment. Another author had utilized the IPION-9 index to measure early orthodontic treatment need in a pediatric dental population in USA and found that moderate and definite treatment needs were seen in 16.36% and 70.91% of children, respectively^17^. A study conducted on Thai population^18^ with high caries prevalence using IPION index had reported that 96% of children aged 8–9 years with mixed dentition needed either moderate or definite preventive and interceptive orthodontic need. Another study in Syrian children^19^ identified that 28.4% and 59.6% of the children showed moderate and definite treatment need respectively, based on IPION-9 index components. To our knowledge, this is the first study conducted among Saudi children using IPION to evaluate the early orthodontic treatment need in the mixed dentition stage and as such, comparison of results from the present study with previous studies was limited.

This study found that according to the IPION-9 scoring system, 59.4% of the sample were in definite treatment need group and only 11.3% of them presented no treatment need. Other authors^17–21^ also have reported high definite orthodontic need in the IPION-9 age group. However, when caries was excluded to detect the impact of other traits, the percentage of sample with no treatment need had increased to 21.3% whereas 34.7% showed moderate treatment need and 43.9% showed definite treatment need. Although the percentage of definite treatment need decreased from 59.4 to 43.9% when the caries scores were excluded, it was still considered very high. Furthermore, in our study, if the moderate treatment need is also considered, 88.7% of the sample were in need of early orthodontic treatment. Majority of the children in this study needed high treatment needs irrespective of the caries component, a finding in accordance with a study on Syrian children^19^.

The high percentage of children with definite treatment need (59.4%) noted in this study could be related to the inclusion of children with permanent incisors erupted in the mouth, regardless of the age, high caries prevalence, and presence of more categories to be scored in IPION-9. The discrepancies between the findings in the studies may be due to the differences in sample selection, ethnic and racial diversity of populations studied, and the index used to assess the orthodontic treatment need. IPION-9 is usually used in children with relatively more advanced developmental age and has more categories and scores when compared to IPION-6. As a result, a higher percentage of sample in this age group may get included in the moderate and definite need categories^19^. Gender comparison showed no statistically significant relation with the treatment distribution needs in the present study (*p* = 0.513), which corroborates with most studies in the literature^5,10,19,22^. Hence, this finding concurs that preventive and interceptive orthodontic treatment needs are irrelevant to the gender regardless of population group. Contrary to this, Dias et al.^23^ found that the males had a need for orthodontic treatment more defined compared to females.

Proximal caries of posterior teeth and early tooth loss are the main components with high importance in IPION as they can result in early space loss due to tipping and/or rotation of adjacent teeth thereby resulting in malocclusion. More than half of the present study sample showed early loss of second primary molars, while 75.4% of children had caries affected first permanent molars. This finding corroborates with that of Almugla^24^ who had reported a high prevalence rate of missing first permanent molars (36.9%) in a study on children from a similar population group in Saudi Arabia and had attributed it to high caries prevalence, except that his study included a wider age group of 7- to 15-year-old children. The goal of this study was to see whether a significant need for early orthodontic treatment existed in the population studied and hence direct comparison of data with other studies is limited. Despite caries still being considered a significant problem in the population studied, a demonstrable need for early orthodontic intervention nevertheless exists even when caries was not counted. Furthermore, it was disturbing to note that children with early loss of primary molars presented no evidence of any space maintenance. This might be attributed to socioeconomic status and lack of awareness of the importance of space maintenance^25^. However, socioeconomic status was not assessed in the present study as a major proportion of the population utilize publicly funded healthcare services.

The IPION-9 can be considered an effective diagnostic tool in that it includes almost all characteristics that indicate the need for preventive and interceptive orthodontic treatment, such as dental crossbite, deep bite, open bite, crowding, missing teeth, and ectopic eruption^26^. In addition, it gives a high weighting score for some components such as proximal caries of second primary molar and first permanent molar compared to first primary molar^1^. However, its limitations are that it does not identify the type of preventive and interceptive orthodontic treatment^15^; radiographs are not assessed which might miss supernumerary teeth and absence of permanent incisors; and absence of permanent incisors is recorded by counting all incisors that are not clinically seen in the arch. Additionally, this index lacks assessment of oral habits and its role in the developing malocclusion. These might affect the estimation of the level of preventive and interceptive orthodontic treatment need^27^, and hence warrants modifications to the IPION to make it more effective and reliable. Rapeepattana et al.^18^ had suggested that grouping the IPION components into a caries and early loss component group, and an occlusal and functional component group, might aid in easy referral by identifying the type of treatment need between the groups. Vakiparta et al.^28^ recommended a policy of initiating early treatment only in subjects with definite need, and to follow-up on those with moderate need and give them time for spontaneous correction. On the contrary, other authors had opined that targeted early orthodontic treatment approach in the mixed dentition would be beneficial and could be considered as an acceptable alternative^29,30^. Reports on early orthodontic treatment need among children during mixed dentition stage is essential for planning treatment strategies as it provides a good juncture to consider interceptive orthodontics^29,31,32^.

One of the limitations in the present study was that the use of population sample derived from children visiting dental health care center in university setting may not represent the general population of similar age group, and hence limits the generalizability of the findings. Since orthodontic care in Saudi Arabia is publicly funded, implementation of early orthodontic treatment could pose a huge challenge. Generating such data could serve as a baseline for future studies involving larger samples and might be useful for planning publicly subsidized orthodontic treatment or could impact policy changes aimed to reduce barriers in accessing and utilizing early orthodontic treatment services at the community and system levels, through allocation of separate funds for the same. This when combined with efforts to increase awareness on the importance of early orthodontic treatment in children could motivate the parents to seek preventive and interceptive orthodontic services thereby improving oral health related quality of life in children and/or parents, and also reduce financial burden on public funded healthcare system in Saudi Arabia.

## Conclusion

This is the first study to evaluate early orthodontic treatment need using Index for Preventive and Interceptive Orthodontic treatment Need in a sample of the Saudi children from the Eastern province of Saudi Arabia. The present study demonstrated that a high proportion of the children in the early mixed dentition presented either moderate or definite preventive and interceptive orthodontic treatment need. There exists a need to educate the parents about early dental visits, provide appropriate anticipatory guidance, and motivate them towards adapting a preventive behaviors.

## Data Availability

The datasets used and/or analyzed during the current study are available from the corresponding author on reasonable request.
